# Factors influencing implementation of the Ministry of Health-led private medicine retailer programmes on malaria in Kenya

**DOI:** 10.1186/1471-2458-10-93

**Published:** 2010-02-24

**Authors:** Yvonne Rowa, Timothy O Abuya, Wilfred K Mutemi, Sam Ochola, Sassy Molyneux, Vicki Marsh

**Affiliations:** 1 Life & Peace Institute, Somalia Programme, PO Box 64495 - 00620 Nairobi, Kenya; 2 Kenya Medical Research Institute/Wellcome Trust Centre for Geographic Medicine Research-Coast, 80108, PO Box 230 Kilifi, Kenya; 3 Health, World Vision, Sudan; 4 Ministry of Health, PO Box 34349-00100 Nairobi, Kenya; 5 Centre for Clinical Vaccinology and Tropical Medicine (CCVTM), University of Oxford, Headington, Oxford, OX3 9DU, UK

## Abstract

**Background:**

Kenya has experienced a number of retail sector initiatives aimed at improving access to antimalarial medicines. This study explored stakeholders' perceptions of the role of private medicine retailers (PMRs), the value and feasibility of programme goals, perceived programme impact, factors influencing implementation and recommendations in three districts of Kenya.

**Methods:**

This study was part of a larger evaluation of PMR programmes, including quantitative and qualitative components. The qualitative research was conducted to assess implementation processes and actors' experiences in the programmes, through focus group discussions with trained PMRs and mothers of children under five years, and in-depth interviews with programme managers, trainers and co-trainers.

**Results:**

PMRs were perceived to provide rapid cheap treatment for non-serious conditions and used as a deliberate and continuously evaluated choice between different treatment sources. All stakeholders supported programme goals and most PMRs described increased customer satisfaction, more rational purchasing of medicine stock and increased medicine sales after participation. Factors undermining programme implementation included a lack of MoH resources to train and monitor large numbers of PMRs, the relative instability of outlets, medicines stocked and retail personnel, the large number of proprietary brands and financial challenges to retailers in stocking antimalarial medicines, and their customers in buying them. Unambiguous national support and a broad range of strategies are important to strengthen the feasibility of change in OTC antimalarial use.

**Conclusions:**

Understanding the context and implementation processes of PMR programmes and the perspectives of key actors are critical to identifying measures to support their effective implementation. Financial barriers underlie many described challenges, with important implications for policies on subsidies in this sector. In spite of barriers to implementation, increased exposure to programme activities promoted trust and improved relationships between PMRs and their clients and trainers, strengthening feasibility of such interventions. Public information can strengthen PMR training programmes by engaging local communities and may facilitate performance monitoring of PMRs by their clients.

## Background

The rationale for encouraging effective home use of antimalarial drugs as a strategy for malaria control is based on an understanding of the importance of self medication, including the popularity of this practice, the role of private medicines retailers (PMRs) in providing medicines used at home, and the inappropriate treatment practices often involved [1-4]. The promptness of self medication practices alongside objectives that childhood fevers are presumptively treated as malaria within 24 hours of onset of illness [5-7] have highlighted the potential role that PMRs could play in malaria control strategies. Whilst concerns exist about regulation of activities in the retail sector, the pragmatic approach of channeling information on Over-The-Counter (OTC) medicines through PMRs has the potential to improve the early treatment of childhood fevers, and may have cost and sustainability advantages over other suggested strategies [8-11]. This is the case in most settings including Kenya where PMRs are licensed to sell OTC medicines such as analgesics, antipyretics, proprietary antimalarial medicines. Although in practice some PMRs include other prescription only medicines in their stock [12,13].

In recent years, Kenya has experienced several private sector initiatives aimed at improving access to antimalarial medicines through the retail sector. The retail sector initiatives in Kenya were based on participatory training of PMRs with public information, and social marketing and social franchising approaches [9,14-16]. The 2001 Kenya National Malaria Strategy recommended such programmes as a means of improving early access to effective antimalarial treatment for children with fever [17]. Between 2002 and 2005, the Division of Malaria Control (DOMC) in the Ministry of Health (MoH) supported over 30 districts to obtain funding through the Global Fund for AIDS, TB and Malaria (GFATM) to implement PMR programmes. On the basis of DOMC implementation guidelines [18], a research team from the Kenya Medical Research Institute (KEMRI)-Wellcome Trust Programme worked in collaboration with the DOMC and District Health Management Teams (DHMTs) in Busia, Kwale and Makueni districts to support implementation and evaluate the PMR programmes implemented.

The current evidence on the impact of the retail sector interventions is largely quantitative [19]. Whereas this evidence is important in understanding the potential role of the retail sector in improving access to antimalarial medicines, it does not provide insights on the dynamics of implementation, actors' perceptions about their role, and the challenges experienced in implementing and practising the intervention. Theory and empirical work around policy considers the importance of implementation processes and their implications for policy achievements. Going beyond conventional public health understandings, the policy literature sees the implementation process as full of contestation, negotiation and bargaining among implementing actors (individuals and organisations), leading to decisions that shape the implementation [20]. The bulk of the current evidence describes the technical content of policy rather than the implementation process and the context in which decisions are made [21].

To address this knowledge gap, this evaluation incorporated qualitative as well as quantitative methods to provide an understanding of the overall effectiveness of MoH PMR interventions in three districts and the factors that influenced this. Findings of the quantitative component have been reported elsewhere [22] and show that the MoH programmes led to a major improvement in PMR practices with a significantly higher proportion of intervention PMRs selling amodiaquine (AQ) medicines with accurate information on its use. There was also an impact on PMR knowledge with more intervention PMRs knowing how to use AQ medicines than those from control areas. This paper aims to describe factors influencing implementation of the PMR programmes in three districts, based on an analysis of qualitative findings.

### Programme Implementation

The programme was coordinated by the DOMC as the lead agency providing technical support for implementation. District Public Health Officers (Officer in charge of control of communicable diseases and general sanitation in the district) together with other district health managers coordinated the programme at the district level. At the divisional level (the fourth administrative tier in Kenya), the Public Health Officers (PHO) were the main trainers. These are officers with two or three years of tertiary level training on the control of communicable diseases in the community and have normal responsibility for regulating the sales of food and medicines in the community, provide licenses to operate, and assess quality through inspecting expiry dates, physical appearances and storage conditions. They were supported by community volunteers, who were selected, either by local chiefs or local communities, from the general community as co-trainers. Co-trainers undertook retailer recruitment, facilitated setting up training venues and conducted public information and monitoring activities alongside MoH trainers.

The core elements of the programmes implemented in all three districts included a five day training of core management personnel; recruitment and training workshops for selected PMRs; development, production and dissemination of information, education and communication materials; public information activities; training of PMRs; and monitoring and supervision activities. Selection of PMRs included all of the following criteria; being the main seller (where more than one seller was present in an outlet); working in an outlet with current stocks of antimalarial medicines; being located in a rural setting; and considered as relatively stable on the basis of local PHO knowledge. PMRs were trained in skill-based workshops by PHOs assisted by community volunteers as co-trainers.

PMRs were invited to a central place in the local settings during normal working hours for a two-day participatory workshop with each workshop having at least 25 participants. The training covered: signs of simple and severe malaria; malaria treatment and prevention; drug resistance; referral practices; storage and expiry of medicines; and communication skills. PMRs were given approximately $ 3.7 (Based on an exchange rate of 1 $= Kes 75) for transport and lunch during the workshops. In Kwale, Busia and Makueni, respectively, 122, 79 and 247 PMRs were reportedly trained in the intervention divisions. In these settings, the number of outlets per division varies between 300-400 retail outlets. Trainings were conducted between December 2004 and February 2005 and the evaluation was conducted about six to eight months post implementation.

Public information activities were conducted by PHOs and co-trainers through public meetings including those of local leaders, church congregations and other special groups, such as women's groups. Public information strategies aimed at creating awareness of the aims of the programme, explaining the roles of PMR and identifying programme outlets. Trained PMRs were monitored through regular shop visits by their trainers (either divisional PHOs or co-trainers) with the aim of continuing education and helping PMRs to solve problems arising from their new practices.

**Figure 1:**
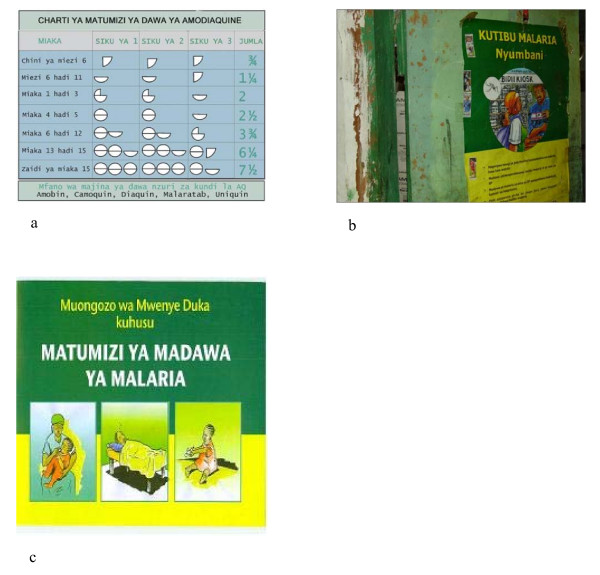
**Samples of materials used for the retailer training programmes in Kenya**: a- is an image of an A4 sized chart for display in retail outlets showing the dose of the MoH recommended antimalarial medicine according to age; b-is an image of a DoMC produced poster to be hung outside outlets to indicate trained status; c- an image of a 24 page booklet for trained sellers covering the main areas of the workshop.

Materials for the programme were either produced locally at district level or commercially printed by the DOMC for distribution to all districts. Locally produced materials for the programme included two A4 sized laminated charts for trained PMRs giving dosage and referral information (Figure 1A). The DOMC produced a poster to be hung outside outlets to indicate trained status (Figure 1B), a 24 page booklet for trained sellers covering the main areas of the workshop (Figure 1C), and a leaflet on the programme for public dissemination. The model for these programmes was based on a generic strategy adopted by the DOMC developed through a series of advocacy meetings by the DOMC and its partners in 2001[23].

## Methods

### Study areas

This study was part of a larger evaluation designed as a cluster randomized trial across three districts with divisions as the units of randomization [22]. The DHMTs in each district identified divisions they considered similar in socio-economic characteristics, malaria burden, malaria control programs and access to health care facilities prior to randomization. In Kwale and Makueni, two interventions and two controls divisions were selected. In Busia, one intervention and one control division were purposively selected. The qualitative component reported here was conducted in the intervention divisions of Kwale (Matuga and Kinango), Makueni (Kathonzweni and Makindu) and Busia (Funyula). Geographically, Busia is located in Western Kenya, with a population of 405,389. Kwale district lies in Coastal Kenya with a population of 544, 468. Both districts experiences year round malaria transmission. Makueni is located in Eastern Province, with a population of 771, 545. It is a semi-arid district with seasonal malaria transmission.

### Data collection methods

These evaluations were conducted between July and October 2005, an average of 6 months for Kwale and Makueni programmes and 8 months for the Busia programme after completion of the last training workshop. Data was collected through focus group discussions (FGDs) targeting trained PMRs and mothers of children under five years of age in the intervention areas. FGDs covered the role of PMRs in treating fevers, training activities and materials used, awareness and support for programme infrastructure and goals, perceptions of feasibility, benefits and disadvantages of using the retail sector channel, perceived impacts, recommendations for various programme activities. In depth interviews (IDIs) targeting programme managers, trainers, co-trainers were used to elicit information on their understanding, perspectives and experiences with the programme. The interviews covered similar issues addressed in the FGDs with additional areas on implementation process factors influencing effective programme outcomes and perceived impacts. Table 1 shows the target groups, methods and number of respondents included in the study.

**Table 1:** Target groups, methods used and number of interviews

Category	Busia (Funyula)	Kwale (Kinango)	Kwale (Matuga)	Makueni (Kathonzweni)	Makueni (Makindu)	Total
Mothers FGDs	4	2	2	2	3	13

Trained sellers FGDs	1	2	1	3	3	10

Trained sellers IDIs	3	-	1	-	-	4

Co-trainers FGDs	1	-	-	1	1	3

PHTs IDIs	1	-	-	1	2	3

Divisional PHO IDIs	1	-	-	1	1	3

Managers IDIs	2	-	-	2	-	4

### Sample selection

Study participants were selected purposively to provide representative views from different geographic areas within study sites. Sampling for FGDs endeavoured to create a homogenous group with similar experiences to facilitate dialogue [24]. Mothers of children under five years were invited from homes near trained outlets. Trained PMRs from rural areas, small and large market centres were invited to attend discussions. Local administrative leaders assisted with recruitment. Each FGD targeted 6-12 participants. For IDIs we aimed to interview all co-trainers, trainers and managers. One research assistant (YR) conducted most interviews supported by (TA) while a locally recruited assistant fluent in local languages took notes. Data was recorded both in written notes and tape recording. Written notes were used to provide backup copies and to capture nonverbal cues. Across all sites an attempt was made to elicit information from different categories of actors involved in the implementation process. In some circumstances a number of actors had been transferred from the districts limiting the number of interviews. However, attempts were made to reach at least all actors available in the district. All discussions were done in languages understandable to the participants and recordings were conducted within the boundaries of confidentiality agreed at the time of discussions.

### Data analysis

All interviews were translated into English and transcribed using Microsoft Word. Illustrative words and phrases were kept in their original language where direct translation was difficult. The interviewer and recorder reviewed information from all interviews at the end of each interview to validate records and to identify new issues. Where relevant, the latter were incorporated into discussion guides for subsequent interviews. Analysis was based on inductive and deductive processes, using a thematic framework [25]. Themes were modified as more issues were identified from the data. Two researchers (YR and VM) jointly conducted the analysis to strengthen the objectivity of the process. Regular consultations were held with other members of the research team to enhance reflexivity.

### Ethical considerations

The study was reviewed and approved by national scientific steering and ethical review committees in Kenya. Data collection was planned around local community timetables and took consideration of events and routine activities. The research aim and processes were explained to all participants as appropriate, and their informed consent was obtained both for participation and for recording of interviews.

## Results

This section presents findings structured around; the stakeholders' perceptions of the role of PMRs, the value and feasibility of programme goals, perceived impact of the programme, factors influencing programme delivery and stake holder's recommendations to improve implementation.

### Stakeholder's perceptions of the role of PMRs

Across all the sites there was consensus amongst users on the general role of shops selling OTC medicines prior to programme implementation. This was described as provision of "first aid", or more convenient access to treatment than expert sources in a range of situations as illustrated by a mother: *"It all depends on the symptoms. At first I will notice the child coughing, then when you touch the head you feel the fever and see whether he has flu so at times like these, I will run to the shop to buy drugs to check on him for about two days. If I see he is not getting better then I will go to the hospital"*

The expert sources referred to were government and private hospitals, clinics and dispensaries. Discussions on the role of general shops referenced relative merits of alternative providers and assumed lack of technical knowledge on illness or medicine use among PMRs. Specific situations were described when hospitals or clinics were far away or closed (evenings, nights, weekends), illustrated by a mother saying that:*"Sometimes you go to the hospital and miss the doctors so you run to the shop as you wait for Monday to see a doctor especially on Sundays." *Retailers are also used when hospitals and clinics do not have medicines available and when visiting clinics and hospitals will involve the mother waiting in queues for a long time or facing harsh staff attitudes as was described by a mother: "*I decided to go to the Government Health Centre where I spent the whole day without being attended to. So when I went back home I decided to purchase malaria drugs from the shops and they got healed. From that experience I decided that I would never ever go back to that Health Centre"*.

The cost of treatment was widely reported as critical in decision-making, with PMRs perceived as offering a more affordable service. PMRs provide commercial services but mothers can choose a drug to fit their budgets, and some PMRs may offer credit to regular customers. These observations mirror studies conducted in other settings where PMRs are patronised due to greater physical proximity compared to health facilities [1,26-29], where carers perceive an illness to be non severe or where outlets are thought to have a more reliable drug supply than clinics [1,30,31]. Similarly, their ability to respond to community demand by selling drugs in accordance with clients' needs [32], being friendly, offering credit to clients and being more affordable than formal health facilities [27] have also been reported as reasons for PMR use.

Specialist drugs shops were a prominent feature of the retail sector in some areas. Earlier surveys in all three districts indicated that PMRs trading from "chemist shops" are found in market centres, particularly in Busia and Makueni [33]. These are registered under the name of a trained pharmacist but may not be run on a day-to-day basis by this individual. However, chemist shops are licensed to sell a wider range of medicines, and dispense these from bulk containers at a cheaper price than the packaged medicines sold in general shops. Many mothers considered chemist shops to be more reliable sources of advice and sell better quality medicines than general shops. The wider choice of medicines, lower prices and perceived reliability also made chemist shops popular. This was not a consistent preference, however, since some mothers were sceptical of chemist shops, recognising the technical limitations of many of the staff employed. A mother described that: "*In some chemist shops, friends, relatives or family members end up serving as attendants. All they do is put on the white coats and then pretend to be qualified to prescribe drugs"*.

General retail outlets were described as having unreliable drug stocks in some instances, especially in the more remote rural outlets, where small shops were reported to carry fewer types of medicines, which were most commonly lower priced antipyretic medicines such as paracetamol and aspirin-containing brands. They frequently did not stock antimalarial medicines, as shown both by local client descriptions and quantitative surveys [22]. This is exemplified by a mother who explained that:*"Not all shops have the drugs. Here we get Malaratab (amodioquine) but in interior places they buy only cheap drugs like panadol and aspirin. They don't buy highly priced drugs." *Larger shops in market centres were more reliable sources of antimalarials. Proximity to a hospital, clinic or chemist also deterred shops from stocking antimalarial drugs in some cases, due to the competitive advantage already described for these providers.

Regarding the interactions between PMRs and clients, the relationship between them was generally characterised by low trust; illustrated by suspicions about over-charging, PMRs' lack of knowledge about health issues, their perceived eagerness to make any sale and the quality of the drugs they stocked. Typically, a PMR described that: *"They (clients) had problems in taking the full dose (of antimalarials). When you tell them to take three tablets then tomorrow take another three... then another... they don't come because they find it hard and think that you want to steal their money"*.

In the absence of any knowledge of the programme, mothers often reported that their own views of PMRs as business people would strongly influence them away from accepting a PMR's advice about drugs. Thus, mothers reported feeling suspicious of PMRs' motives if they recommended that customers buy medicines other than those requested. This concern about profit motivation was reinforced by the experience of different shops selling the same medicines at different prices. In addition, prior to the programme, the vast majority of clients did not consider PMRs to be good advisors on health issues. PMRs themselves reported reliance on package instructions, their own experience gained from trial and error, observations of treatments used in clinics and mass media advertising for information on use of medicines. Since most medicine packages in Kenya contain information on active ingredients and doses, less literate PMRs (and mothers) were felt to be particularly disadvantaged by being unable to read package instructions. For these reasons, and as reported elsewhere [8,34], choice of OTC medicines was therefore largely demand driven.

PMRs described the same phenomenon, explaining that they sell the medicines customers ask for, and describing customers as being unprepared to consider alternatives. Both PMRs and their customers often complained about each other's attitudes, PMRs being described as untrustworthy, arrogant and rude and customers as argumentative and not prepared to listen. One PMR reported that: "*There is a group of customers who are stubborn to a level that whenever they come to the shop and order for two Aspirins, two Panadols and one Action tablet they just expect to get that and no more questions or instructions. They may even become abusive if you tried telling them that you feel that they would better take antimalarial drugs. With such people we just give them what they ordered for and business continues"*. Mothers also described concerns about the quality of drugs in general shops and the effect of the way they are stored and handled on this. Although the quantitative assessment showed that antimalarial medicines were usually sold within their expiry dates [33], it was widely felt that PMRs might be selling expired drugs. There was a special concern reported on unpackaged drugs sold loose from tins, where information on the outside of the tin might not correspond with the contents. This compounded with the fact that in Kenya, medicine packs do not contain any indicator of registration status from the regulatory authority, makes it difficult to track quality of drugs sold. In addition, mothers believed that storing drugs in positions where they would be open to the atmosphere or exposed to dirt, smoke and sun would affect their quality.

From this description, it emerges clearly that the commonly perceived role of PMRs is to provide a pragmatic solution for clients who are seeking rapid and affordable treatment for conditions that are not thought to be very serious, a choice highlighted by dissatisfactions with and inaccessibility of formal sector providers. Their use is a result of a deliberate and continuously evaluated balance of choice between different potential treatment sources. Major reservations with PMR services concerned their commercial orientation and lack of expertise in health care (from clients) and their unregulated and fluid nature (from trainers and managers). These factors have a major influence on the relationship between PMRs and their clients and potential trainers, which in turn forms the backdrop to any intervention targeting behaviours of these key actors.

### Value and feasibility of programme goals

Across all groups, actors supported programme goals. Amongst the mothers who participated in FGDs, awareness of the programme followed the same patterns as the intensity of public information and PMR training, with greater awareness in those areas where more activities had taken place. However, while largely expressing support for the programme goals, many mothers expressed reservations about feasibility. A strong sentiment concerned the influence of the personality and attitude of individual PMRs, thus: "*It depends on a person's attitude ....there are people who may have a negative attitude and will not bother or have time for you while others will have a positive attitude and are committed regardless of any other issues like time"*.

Direct experience and prior awareness of the programme appeared to affect the way that mothers discussed both the sector and the programme. Positive ideas about retailers and fewer reservations about the feasibility of change were expressed in the groups with prior awareness or direct experience of interacting with a trained PMR. This change in attitude - and its cause - was also illustrated by a PMR saying that:*"The seminar was good because I had difficulty telling people about it but (later) they would come and tell me, 'we have heard that you were trained on how to use antimalarial drugs, now I need them*". Programme materials were considered particularly important by PMRs, who frequently described programme drug dosage charts and posters as acting as "certification" for the training. PMRs described some additional factors they believed promoted change as age, with younger customers showing more flexibility, and customers' previous experiences with drug advice from trained PMRs. Mothers' support for the programme goals was described in terms of improving the health of the community; reducing malaria; improving the way that retailers sell and advise about drugs; improving knowledge of the community about OTC medicines; using OTC medicines better; improving access to treatment; and saving money by using correct medicines in the right way.

Trained PMRs expressed clear and supportive ideas about the rationale for the programme around improving quality of care given: *"The organizers realized that some community members treat malaria by taking the wrong dose. They also buy drugs from shops that could be poisonous to them. So they found it better to train the PMRs so as they can train others on how well to use the drugs"; *information on new drug policy changes *"We were taught so that we can move with the times and not just continue with sulphadoxine pyrimethamine (SPs) yet the drug at the moment is amodiaquine"; *and knowledge on malaria danger signs: *"They felt the ones that are serious or have danger signs should go to the hospital and the ones with minor symptoms to be treated in the shops."*

Given their profit orientation, it was important for implementation that a majority of PMRs felt that they had benefitted financially from participation. The key content issue that was raised concerned the antimalarial medicine promoted through the programme, including confidence in the programme drug, confusion over its identification and use and concerns about unexpected adverse events. A major contributor to these concerns was the delay and confusion at national and district levels in identifying the most appropriate OTC antimalarial medicine, given that these programmes were initially rolled out at a time of national antimalarial drug policy change. An interval of over one year elapsed before beginning sensitisation for this programme within districts and eventual rolling out to PMRs. The drug that was finally identified, amodioquine, had been available in private retail outlets for some time, and was not therefore a new medicine. However, a total of 12 brands of amodioquine were found in shops in each district, making it difficult to identify which antimalarial medicines were amodioquine. In this commercial market, promotional materials could not be developed for specific brands and there was no policy on over-branding to support product recognition. This led to difficulties for trainers, PMRs and their clients in consistently identifying amodioquine medicines at retail outlets. Confusion over brand names, regulatory infringement and poor quality drugs have been concerns for the inclusion of the retail sector in malaria control in other settings [13,19,32]. The quantitative surveys in this programme evaluation [22] also pointed to confusion between different types of antimalarial brands. Overall, the number of brand names in the market continued to challenge the familiarity of both PMRs and their clients on the nationally recommended antimalarial medicines especially during this transition period of drug policy changes.

Discussions with many PMRs illustrated that they are often challenged by the conflict between profit motivation and a real desire to offer a good service to the community. This may reflect the fact that the study was largely conducted in rural areas, where PMRs would often be neighbours or relatives of many of their clients. An interesting aspect of this relationship was the practice of offering credit to some clients as a coping strategy to increase demand for high cost of antimalarials. PMRs often discussed providing credit as a practice carrying high risks which they preferred to avoid unless customers were well known and had a good track record of repaying debts. Their more likely response to a mother with insufficient funds would be to recommend alternative medicines. However some found it morally difficult to sell medicines that they knew - after the training - were relatively ineffective, saying for example: "*There's a negative experience. When someone comes to buy SP, I find it hard to sell to him. So the stock is still there and that's a loss to me"*. Others described that they would provide credit to less well-known customers in extreme circumstances, for example serious illnesses or where there is no means of travelling to a clinic. One PMR described losing money through credit as inevitable, saying that: *"One customer will come and say 'I am sick and I don't have a shilling' so you give out drugs on credit then he brings you the money later so you help one another. But for others when you give them it's like you have started a war... so he will run away and never come back, and when he gets money he runs to another shop"*.

### Perceived impact of programme

A summary of the perceived impact of the programme and moderating factors are presented in table 2. All groups reported improved knowledge and practices on the use of OTC antimalarial medicines as a result of the programme. PMRs consistently reported improvements on giving advice to customers on medicine use. This was often confirmed by mothers and trainers, particularly in the district with intensive implementation. Other changes described by PMRs were referring clients who were very ill to hospital; not selling medicines to very young children; knowing how to treat own family members; selling more medicines; getting more customers and making more profits. For example, PMRs that already stocked antimalarials were sometimes able to persuade clients to buy full courses rather than single doses. This led to reported financial benefits attributed to increased turnover of drug stock due to selling full instead of partial courses as illustrated by a retailer: *"Our profits depend on how drugs move... before you could sell one box per month but now you sell 3 boxes in a month. If for one box you get a profit of 50 shillings in a month you will have 150 shillings"*. Some trained PMRs reported savings through greater efficiency in stocking antimalarial and antipyretic drugs as a result of increased understanding of the differences and similarities between them. In addition some reported improved status and relationships with customers.

**Table 2:** Summary of reported impacts, and perceptions of moderating factors

Reported impacts	Moderating factors
**ON COMMUNITY:** Positive attitudes/better relationship with PMRs Improved use of OTC medicines/some report no change* More likely to accept advice from PMRs Experienced better outcomes from treatment	*Positive*: Knowledge of programme (through public information or direct experience) Availability of programme materials (act as validation of training) Shopkeeper's knowledge, attitude and communication skills Availability of credit *Negative*: High cost of drugs Lack of familiarity with drugs/preferences Side effects of drug Inability to read instructions Time taken in buying drugs from trained retailers Use of proxy customers Rapid improvements in symptoms Contradictory messages on packaging and mass media (e.g. radio advertisements)

**ON PMRs:** More knowledge about malaria and its treatment More likely to ask customers questions, give advice on drug use and refer to hospital Financial gains from selling more drugs and getting more customers Financial losses if not able to sell Improved status in community Better relations with community and public health officers Less likely to sell expired drugs†	*Positive*: Exposure to training Materials: support training and validate trained PMRs Public information activities (where held) Shopkeeper's attitude positive Customers' attitude positive High coverage of shops in surrounding area *Negative*: Cost of drugs Instability of some shops and staff in shops Proximity to hospitals, clinics and chemists (don't stock antimalarials because cannot compete on prices) Situated in remote rural areas: often small outlets, do not stock antimalarials because expensive to stock and low turnover Time taken in training and advising

**ON TRAINERS AND MANAGERS (Busia and Makueni‡)** More knowledge and skills on malaria control, training and programme management Improved status for trainers in community and with PMRs Better relationship between PHOs and PMRs PHOs supported to conduct other routine activities Trainers feel positive - bringing about change and getting positive feedback from PMRs	*Positive*: Positive attitudes in DHMT Technical support from KEMRI *Negative*: Inadequate resources such as coverage of programme, unable to undertake monitoring and public information activities, low allowances to co-trainers, materials not adapted to current drug policy Delays in disbursement of funds

* No change especially reported by mothers in Kwale

† This perception was reported by mothers

‡ Trainers and managers not interviewed in Kwale

Another perceived impact was the improved relationship between PMRs and their regulators especially in the district with highest levels of implementation. Their roles had reportedly led in the past to tensions between PHOs and PMRs. However, many PMRs spoke in positive terms about their trainers' skills and attitudes during the workshops. A majority welcomed the idea of monitoring visits and described feeling free to ask questions. This suggests the potential for an outcome of improved relationships, resulting in PHOs having greater influence and more opportunities to further strengthen PMRs' practices. This would be of particular importance during drug policy change, including the introduction of artemsinin combination therapy (ACT) in this sector. It may also support greater involvement of PMRs in other public health programmes with impacts beyond the selling of antimalarial drugs. Of importance for sustainability, involvement in the programme and observation of an impact of their efforts were valued by many trainers, particularly in the district with highest levels of implementation and PMR behavior change. For example, one such trainer reported: *"I was pleased because there is a place I had gone to visit shops and there was a mother who was being sold drugs for a child. The seller asked her questions and I found that really what I had trained was being followed. I felt happy"*.

### Factors influencing programme delivery

The barriers and facilitating factors for changes were reported consistently across all districts by all actors. A majority of mothers reported that the main drive to change at community level was knowledge of the programme itself, through public awareness activities and through direct contact with trained PMRs and programme materials. Most PMRs on the other hand perceived the training itself as the main factor facilitating their own change, strongly supported by monitoring and public information activities and programme materials.

Positive attitudes and support from DHMT were reported as important factors supporting change within the training teams, but trainers and managers generally discussed a lack of resources to fully implement programme activities as a major limitation. For some trainers and most co-trainers, this primarily concerned the lack of allowances for transport and undertaking training or monitoring activities. For the managers, the main budgetary limitations were in supporting adequate public information and monitoring for the programme, where these were seen as key to change for trainers, PMRs and their customers.

Several factors were widely discussed by mothers, trainers and managers as major barriers for change. For example, the time taken for a trained PMR to ask questions and offer advice reportedly dissuaded customers from using the outlets either for their own drug purchases or while they waited for others to do the same. In Busia, managers described an additional challenge as the relative instability of shops and trained personnel within shops. Their experiences had shown that many shops open and close repeatedly over time, depending on their economic circumstances and availability of personnel. Many employees in shops had a rapid turnover as they moved on to better paid jobs. This emphasised the need for repeated training and for staff to be able to conduct on-the-spot training during monitoring visits.

Regarding the training itself, the direct and indirect costs of attending workshops and stocking recommended drugs were important barriers. Attending workshops could involve losses due to closing an outlet during training and spending cash on transport to the training venue. These were immediate challenges for families relying on petty trading to buy food and other essentials on a daily basis. In practice, the turnout of PMRs at workshops was relatively good with over 80% of invited PMRs attending training and with a consistent pattern of increasing turnout as the programme became more established in all districts. However, despite some PMRs reporting increased turnover of drugs (as was mentioned earlier on) others, especially those who did not regularly stock antimalarial medicines prior to training, found it difficult to afford them in the same way that their customers reported difficulty in purchasing these. Since their practices are largely led by consumer demand, and as their small-scale businesses generally operate on low profit margins, they tended generally to stock goods with a rapid turnover. Overall, PMRs faced challenges in selling drugs especially the expensive drugs a problem particularly faced by those in remote rural areas and those near to clinics, pharmacies and hospitals.

Factors that limited the likelihood of customers buying amodiaquine in full doses tended to deter PMRs from stocking and recommending them. This includes both the cost and any perceived side effects of the drugs. A particular cost disincentive was experienced by one group of PMRs in Makueni who reported that they had stocked amodiaquine drugs after the training but had been unable to compete with the lower prices (and perceived greater reliability) of the nearby chemist and hospital, leading to unsold stock. In all districts, some retailers reported being unable to afford to stock amodioquine, and these individuals did not perceive financial gains from the training. Where sellers felt unable to benefit financially from the training, they were much more likely to complain about the costs of participating in the programme and to demand benefits, such as better compensation for their time during training or the provision of starter packs of drugs. Where sellers described increased profitability of their businesses in the longer term as a result of the training, this seemed to provide significant compensation for any losses incurred during the workshop in all districts.

The instructions on amodiaquine use by age developed by the DoMC [35], and provided to trained PMRs as a reference material for the programme, produced a practical anomaly given the manufacturers' "blister pack" packaging style and instructions for this drug. Following DoMC recommendations meant that clients would always have to purchase more pre-packed amodiaquine than the recommended dose at every age. PMRs were advised to recommend that their clients dispose of left-over portions of tablets, which could be up to one and a half tablets for a full adult course. This was an unpopular recommendation for families buying expensive medicines and experiencing frequent episodes of fever. In addition, most manufacturers recommended a different dosage to the government recommended regime, an observation that was reported as leading to confusion amongst both PMRs and their customers. Some trainers described being confused by hearing contradictory drug dosage messages as part of radio advertisements promoting SP drugs. Finally, some actors described side effects of amodiaquine as being barriers to their use, and where this occurred the experience could be marked.

All trainers described frustrations concerning rumoured expectations of benefits during programme implementation. For example, many co-trainers became frustrated when expected benefits, such as reimbursement for time spent outside the workshop and provision of bicycles to support travel, did not materialise. The allowances paid during the workshop were also generally considered inadequate, especially for those who had to remain away from home overnight. In both Makueni and Busia, confusion about allowances led to rumours that the funds originally allocated to them were being used for other purposes. Similarly, some co-trainers had questions about the accounting system for allowances paid during the workshops. Some believed that they had not been sufficiently consulted in planning the trainings, giving an example of being informed last minute about workshops. One group described their "powerlessness" to act in support of perceived problems, fearing that they would not be retained within the programme. These frustrations and lack of support for transport undermined co-trainers ability to participate actively in the programme. These issues illustrate some of the difficulties associated with programmes incorporating community "volunteers" described in other settings [36]. In this programme, these difficulties were accentuated by unmet expectations arising from lack of clarity about tasks, roles and availability of re-imbursement. The choice of personnel was important. Interviews with volunteer co-trainers in the district where selection was made by the local community suggested that they accepted the volunteer role and reported higher levels of job satisfaction than in districts where chiefs were largely responsible for the appointment. This observation concurs with recommendations that interventions with wider buy-in are likely to be more successful [19].

### Stakeholder recommendations to improve implementation

Strong central support emerged as a critical issue in all districts. All stakeholders expressed opinions on areas where more national level strategic support was needed, including the overall approach of working with medicine retailers, national coordination of information on medicine use, provision of adequate programme resources and tackling affordability issues.

The selection process for PMRs in this programme was a challenging aspect of implementation with both positive and negative outcomes perceived for any given strategy across actors. In principle, the programmes aimed to train all retailers selling antimalarial drugs, but, in practice, this proved a very difficult criterion to define. One issue was the changeable nature of drug stocking practices, with fluctuations being largely dependent on the PMR's current financial status; small rural shops being particularly vulnerable to this effect. In any case, the large numbers of such outlets made it difficult for managers to collect accurate information on shop status, including drugs stocked, at any given time. In this study, there was no consensus amongst stakeholders on which shops should be included in the programme.

## Discussion

This paper has illustrated the importance of understanding factors underlying implementation in assessing PMR, and other, programme impacts. The data corroborates quantitative outcomes and provides a deeper understanding of some of the potential influences over effectiveness of these MoH interventions. It emerges from this evaluation that the proposed role for PMRs in malaria control, of acting as advisors and promoting a public health good, is in conflict both with their normal practices and with mothers' perceptions of their underlying motivations. The qualitative findings are complex; some point to the feasibility of change, and others at significant barriers requiring programme modification before either retailer or community OTC medicine use can improve on a wide scale.

One potential limitation in interpreting this data is the positionality of the researchers (from KEMRI), since this organization was often perceived as having played a significant role in the supporting with MoH in designing the programme activities. There may therefore have been a tendency for some interviewees, particularly district managers and trainers, to focus more on positive than negative aspects of the programme. To counter this effect, researchers endeavored to set up an open and non-judgmental atmosphere for the discussions and explained the importance of being open about views so that we could learn from their experiences. The second limitation regards period of evaluation (6-8 months post implementation), making it difficult to assess the likelihood that the positive impact of the intervention would be maintained over time. This is also compounded by the fact that there were limited supportive supervisory activities conducted.

### Barriers and facilitating factors for programmes

Some findings underline described barriers to change in PMR programmes, including the resources needed within over-stretched government health services to train and monitor large numbers of largely unregulated medicine sellers [8,10,37], the relative instability of outlets, medicines stocked and retail personnel [9], the large number of proprietary brands, the substandard quality of some OTC medicines [10,38], and the wide availability of counterfeit medicines. Further important challenges to emerge from this study are the lack of trust of customers towards PMRs; the resulting reluctance to accept advice to buy a different, more expensive medicines; and the economic challenges to retailers in stocking antimalarial medicines, as well as to their customers in buying them. At a more central level, the study strongly emphasises the need for unambiguous support and a broad range of national strategies to strengthen the feasibility of change in community use of OTC antimalarials [8,39,40], including: provision of a clear drug policy for the sector; central coordination of drug use information; adequate drug quality control; and strategies to support economic access to full courses of antimalarial medicines.

Despite these challenges, there are several findings that support the feasibility of such programmes promoting change. As a fundamental starting point, all stakeholders perceived the programme goals to be valuable. Furthermore, given the commercial nature of a retailer's role, it is crucial that the majority of retailers reported positively on the programme's impact on their businesses, describing increased customer satisfaction, more customers, more rational purchasing of medicine stock and increased medicine sales. The overall improvement in the relationship between retailers and their clients that was described by some may also be important to counter the mutual mistrust that commonly characterises their normal relationship. It is of interest that improvements in the relationship, and increased confidence amongst retailers about the medicines they sell, may encourage retailers to extend credit facilities to their customers. This could be an important step in supporting better access to effective malaria treatment for the poorest community members. Customers with experience of buying medicines from a trained retailer reported markedly better attitudes towards the sector than those without, although still recommending greater community involvement in such programmes. This would suggest that over time, if the programme is successful, peoples' concerns about shopkeeper attitudes might diminish. This conclusion is supported by the increased programme impact and more strongly positive stakeholder perceptions for the programme in the district with highest levels of implementation. But many of the comments made by mothers illustrate that this had already begun to happen to some extent in all districts. This strongly supports ideas that measures that build trust in trained PMRs from the outset are critical factors for success. This is perhaps the most important area that programmes currently do not address; that is, the relationship between medicine sellers and their clients, and the need to build trust between these groups, for example, by greater community involvement, public information and monitoring activities.

### Which types of outlets should be included in PMR training programmes?

Criteria for selection of PMRs for the programmes were a debated issue amongst stakeholders, with advantages and disadvantages described across three main different approaches: targeting all outlets, targeting outlets selling antimalarial medicines and targeting highly used outlets. The first approach was mainly recommended by retailers who were clear that as many shops should be included as possible to reduce the chance that customers would use non programme shops and therefore reduce any potential competition between trained and untrained PMRs. In addition, their descriptions of practices within the sector made it clear that outlets cannot be easily separated into those that stock antimalarial drugs and those that do not as this often varies, especially in the smaller more rural outlets. Mothers strongly supported the second approach of training all outlets selling antimalarial medicines, based on their interest in a broad regulatory function for training. Some managers and trainers identified the third approach as a pragmatic way of maximising efficient use of resources by targeting fewer, larger, busy shops selling antimalarials in market centres. Others supported PMRs' concerns that selecting specific shops could create conflict between trained and untrained retailers.

These three selection approaches can be seen to fit with different strategic models for drug retailer programmes [7,41]. Targeting all outlets selling medicines is clearly a huge task, and unlikely to be manageable within the MoH using a participatory training and monitoring approach as represented by this programme. Programmes with similar coverage aims have achieved this through mass communication and social marketing approaches [14,42]. By ensuring an informed public, mass communication approaches may enable customers to directly regulate or at least check on sellers' activities. The model of targeting highly used outlets selling antimalarials leaves many opportunities for community members to purchase medicines from non-programme shops. Not surprisingly, retailers were wary of this model. A highly selected, small group of trained outlets are only likely to have a broad impact on community drug use if these shops are very strongly promoted and given a competitive edge, with implications for the level of monitoring needed to ensure that an adequate quality of care is given. This approach is close to a social franchising model, involving a closely regulated relationship between the MoH (franchisor) and outlets (franchisees) with widely promoted subsidised products. In the social franchise model, the MoH would take responsibility for protecting the community from potential manipulation for profit by highly trained retailers. This type of model has been suggested for shops as a potential strategy for distributing newer, more expensive and complex antimalarials (ACTs) alongside rapid diagnostic testing [43]. It is also currently being implemented in Kenya for community health worker outlets and in Tanzania for a subgroup of shops registered to sell antimalarials [44]. The middle approach, supported by mothers and some trainers, targets as many shops selling antimalarials as possible. Given the inevitable resource limitations for monitoring and the need to address the relationship of mistrust between sellers and their customers, investing in public information could support the over-stretched government regulatory systems and track local contextual issues for the programme. This approach requires that resources are shared more evenly between training retailers and strengthening community involvement, maintaining a balance of knowledge, and therefore power, between them. The programmes evaluated in this study lie close to this latter approach, with potential for greatly increasing impact and sustainability by strengthening the role of the local community in planning and monitoring activities.

### Can PMR training programmes generally promote better use of OTC antimalarials?

The main rationale for optimising PMR practices within a malaria control strategy lies in the increased geographic (and therefore, indirectly economic) access that shops provide in rural areas. However, these findings point to a paradox for access and availability of OTC antimalarial medicines since it appears that areas with the greatest challenges in accessing these medicines (that is, remote rural areas) are also the ones served by outlets that are least likely to stock antimalarial medicines. This reportedly occurs because neither the PMRs nor their customers can afford to buy these relatively expensive drugs. This finding is consistent with other reports on drugs stocked in private retail outlets [9,10]. Some outlets in rural areas also experienced a similar economic challenge in stocking antimalarial medicines since they were in direct competition with either chemists selling loose tablets at a lower price or clinics offering subsidized services. Many retail outlets were therefore unable to make the switch to stocking antimalarial medicines, both because they cannot afford to purchase the stock and because their clients cannot afford to provide a regular demand for them. Such observations provide further evidence for the need to provide subsidies in the private retail sector for expensive medicines, and will be of critical importance in the introduction of ACTs to peripheral level distributors [44].

## Conclusions

This study describes the experiences, perceptions and attitudes of stakeholders in three PMR training programmes in different districts in Kenya. The findings were similar across districts, but with important additional supportive findings from stakeholders in one district where greater levels of implementation occurred. The findings show that stakeholders were generally supportive of programme goals but that the proposed role for PMRs, of acting as advisors and promoting a public health good, was generally in conflict both with their normal practices and with mothers' perceptions of their underlying motivations. Some findings point to the feasibility of change and others at significant barriers requiring programme modification before either retailer or community OTC medicine use can improve on a wide scale. Understanding the context and implementation processes of PMR programmes and the perspectives of key actors are critical to identifying measures to support their effective implementation. In practice, most PMRs described increased customer satisfaction, more customers, more rational purchasing of medicine stock and increased medicine sales after participation. Public information can strengthen PMR training programmes by engaging local communities and may facilitate performance monitoring of PMRs by their clients. Factors undermining programme implementation included a perceived lack of central support for the approach, lack of MoH resources to train and monitor large numbers of PMRs, the relative instability of outlets, medicines stocked and retail personnel, the large number of proprietary brands and financial challenges to retailers in stocking antimalarial medicines, as well as to their customers in buying them. Financial barriers underlie many described challenges, with important implications for policies on subsidies in this sector. However, increased exposure to programme activities promoted trust and improved relationships between PMRs and their clients and trainers, strengthening the feasibility of such interventions.

## Competing interests

The authors declare that they have no competing interests.

## Authors' contributions

YR- was involved in designing and coordinating the study, collected the qualitative data, conducted the first analysis of qualitative data, drafted the initial report and contributed to the drafting of the manuscript. TA- was involved in designing and coordinating the study, contributed to data collection and data analysis, and drafted the paper. WM- was involved in the conceptualising the design of the study and supported data collection process. SO- contributed to conceptualising the study and coordination at central level. SM- Contributed to the tool development, supported data analysis and interpretation. VM- Was overall responsible for the design and supervision of the study,, supported data collection, analysis and interpretation and oversaw the overall intellectual content of the manuscript. All authors read and approved the final manuscript.

## Authors' informations

At the time of the study: YR was working with KEMRI CGMRC as research assistant while WM was working for the Ministry of Health in the Division of Malaria Control coordinating retail sector activities. YR is now working as a Technical Advisor, Life & Peace Institute, Somalia Programme. WM moved to World Vision, Sudan programme to coordinate malaria control activities. SO was the Manager of the Division of Malaria Control, Ministry of Health and he now heads health services within Nairobi province, Kenya.

## Pre-publication history

The pre-publication history for this paper can be accessed here:

http://www.biomedcentral.com/1471-2458/10/93/prepub
